# Camera-Based Monitoring of Neck Movements for Cervical Rehabilitation Mobile Applications

**DOI:** 10.3390/s21062237

**Published:** 2021-03-23

**Authors:** Iosune Salinas-Bueno, Maria Francesca Roig-Maimó, Pau Martínez-Bueso, Katia San-Sebastián-Fernández, Javier Varona, Ramon Mas-Sansó

**Affiliations:** 1 Department of Nursing and Physiotherapy, University of the Balearic Islands, Health Research Institute of the Balearic Islands (IdISBa), 07122 Palma, Spain; iosune.salinas@uib.es (I.S.-B.); paz.martinez@uib.es (P.M.-B.); 2 Department of Mathematics and Computer Science, University of the Balearic Islands, 07122 Palma, Spain; xavi.varona@uib.es (J.V.); ramon.mas@uib.es (R.M.-S.); 3 Department of Nursing and Physiotherapy, University of the Balearic Islands, 07122 Palma, Spain; katia.sansebastian@uib.es

**Keywords:** camera-based, inertial sensors, neck, cervical rehabilitation, serious game, head-tracker, therapeutic exercise

## Abstract

Vision-based interfaces are used for monitoring human motion. In particular, camera-based head-trackers interpret the movement of the user’s head for interacting with devices. Neck pain is one of the most important musculoskeletal conditions in prevalence and years lived with disability. A common treatment is therapeutic exercise, which requires high motivation and adherence to treatment. In this work, we conduct an exploratory experiment to validate the use of a non-invasive camera-based head-tracker monitoring neck movements. We do it by means of an exergame for performing the rehabilitation exercises using a mobile device. The experiments performed in order to explore its feasibility were: (1) validate neck’s range of motion (ROM) that the camera-based head-tracker was able to detect; (2) ensure safety application in terms of neck ROM solicitation by the mobile application. Results not only confirmed safety, in terms of ROM requirements for different preset patient profiles, according with the safety parameters previously established, but also determined the effectiveness of the camera-based head-tracker to monitor the neck movements for rehabilitation purposes.

## 1. Introduction

Camera-based interfaces provide a hands-free approach to interact with devices. Therefore, they have a straight application in assistive tools for motor-impaired users [1] but also for rehabilitation purposes [2]. Specifically, head-trackers vision-based interfaces, a camera-based approach that tracks the motion of the head, could be used for neck rehabilitation purposes as they demand the movement of the neck joints to interact with the device.

Neck pain is one of the most important musculoskeletal conditions in prevalence and years lived with disability, as it becomes chronic in 30–50% of cases [3,4]. The common treatment of neck pain includes conservative and pharmacological interventions. Conservative treatments often include therapeutic exercise, with the aim of improve mobility, pain, function and quality of life [5,6,7]. Therapeutic exercise, usually supervised by a physiotherapist, includes mobility, strengthening, endurance and motor control exercises [5,7,8]. The long-term beneficial effect is achieved by performing the therapeutic exercise constantly and over time, which requires high motivation and adherence to treatment, especially when it is done at home. The adherence to the treatment will therefore be essential for the maintenance of the effects [5]. One potential way to increase adherence is by means of an exergame, that is, a serious game that aims at physical exercise (in our case, neck therapeutic exercise) while entertaining the user. The exergame allows therapeutic exercise performance with assets of motivation, supervision and feedback, which are key factors of adherence [9,10].

With that aim, we started the design and development of a specific mobile application that monitors and analyses the fulfilment of neck therapeutic exercises at home (or elsewhere). The application includes an exergame individually adapted to the user capabilities at any time of the rehabilitation process. By means of a camera-based head-tracker, user head motion is detected, and used to interact with the exergame application in order to monitor that users perform correctly the neck movements. The use of the camera has the advantage that it is not invasive, and the user can move comfortably without external limitations. In addition, the fact that it is a mobile application allows doing the therapeutic exercise, with supervision and feedback, available everywhere, anytime, and to anyone.

This paper presents the validation experiments performed in order to (1) explore the feasibility of using the head-tracker interface for neck rehabilitation purposes, in the sense of the range of motion (ROM) that the head-tracker was able to detect and (2) determine the safety of the application, as an exploratory safety experiment, in terms of neck ROM solicitation by the mobile application.

### 1.1. State-of-the-Art

It has been proved in the literature that introducing gamification to therapeutic exercise in rehabilitation increases its effectiveness and adherence [11,12,13,14]. In this sense, serious exergames have been explored for different rehabilitation purposes, both using commercial games and devices in a rehabilitation context [15] or designing specific serious exergames for health and rehabilitation purposes, under more specific and clinical criteria design [11,12,13,14]. Serious exergames have proved to be effective in different neurological and musculoskeletal diseases [12,16,17]. In recent years, several virtual reality (VR) (immersive and non-immersive) serious games for rehabilitation have been developed [16,17,18,19]. If they were specifically aimed to a body part, they needed to attach a wearable sensor to the part of the body, as happened in the work of De Oliveira et al. [17], Rechy-Ramirez et al. [20] or interact with a full body camera detection [21]. With the aim of using serious gaming with smartphones or mobile devices, with integrated sensors on the mobile phone, Baranyi et al. [16,22] are developing a serious game system for post-stroke rehabilitation. Regarding specifically neck region, Mihajlovic et al. [23] developed a virtual reality serious game, but in order to create the immersion on VR a headset needed to be used by the patient. As for neck movements detection with mobile devices, there have been some authors that have developed neck exercise systems with sensors integrated in mobile devices, i.e., with camera-based head tracking. Lawanont et al. [24,25] developed a neck movement image detection integrated in the smartphone sensors. It was able to calculate neck flexion, so it fulfilled the purpose of monitoring head posture while using smartphones. Yet, it did not detect or monitor other directions of movement. Thus, the issue of detecting all directions on neck movements with no sensors on the user, and to use them as an interaction for a serious exergame for neck rehabilitation with a mobile device remains to explore.

### 1.2. Camera-Based Head Tracking

One common sensor on smartphones and tablets is the front-facing camera, that can be used to perform the user’s head tracking. Such camera-based head-trackers provide a way to interact with these devices through the movements of the head. Using a vision-based head-tracker has several advantages. Mainly, it does not require any calibration or additional hardware, apart from the components already built in mobile devices. It also has the additional advantage that it is not invasive, i.e., the user can comfortably move without the burden imposed by wires or markers. The only requirement is that the main structures of the face (eyes and nose) have to be fully visible by the front-facing camera.

We have previously developed a camera-based head-tracker for mobile devices [26] that automatically detects and tracks the position of the user’s nose. To track the nose, the system extracts facial features of its surrounding region, and returns the average of all these features as the nose point. As this region is never occluded by facial hair or glasses, it can be continuously tracked despite the orientation of the user’s head when he or she is looking at the screen. We direct the reader to Roig-Maimó et al. [26] for a technical description of the head-tracker interface.

Previous work validated the head-tracker’s viability as a pointing device in target-selection tasks with able-bodied [27] and motor-impaired users [28]. Once the head-tracker interface was validated from the point of view of Human-Computer Interaction, in this work we analyse the feasibility of using it to develop applications for cervical rehabilitation purposes, as it allows the interaction with devices through the movement of the head.

## 2. Exploratory Experiment: Neck Range of Motion Tracked by the Camera-Based Head-Tracker

An exploratory experiment was conducted in order to explore the neck ROM that is effectively tracked by the camera-based head-tracker. With this experiment, we wanted to determine at what extent the head-tracker was able to track the mobility range of the neck, in order to determine if it could be used for ROM rehabilitation purposes.

**Figure 1:**
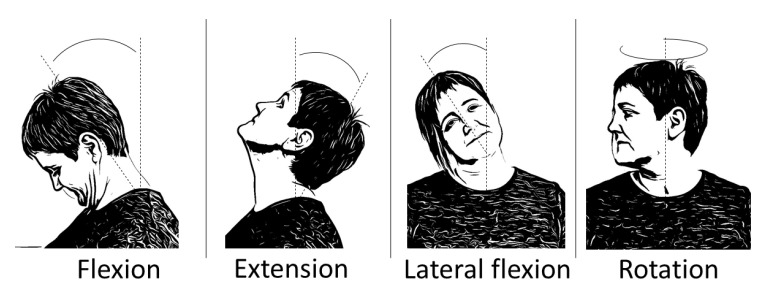
Movements of the neck.

The cervical area has a normal maximum mobility of 45${}^{{}^{\circ}}$ of flexion, 45${}^{{}^{\circ}}$ of extension, 45${}^{{}^{\circ}}$ of lateral flexion (each side) and 70${}^{{}^{\circ}}$ of rotation (each side) [29]. See Figure 1 in order to observe the movements of the neck joint. Functional mobility, understood as the range of motion used in daily life activities, is 20% to 40% of maximum available cervical ROM [30,31]. Any kind of change in those ROM would alter the functional mobility of the neck and spine, which could alter also the functionality of individuals, causing disability movement [32,33].

Seven participants (six females) were recruited from a university campus in Spain. They were staff from the university campus who volunteered for the experiment. Ages ranged from 29 to 48 with a mean of 39.14 years (SD = 6.76). There were no requirements or exclusion criteria on prior experience to participate in the experiment.

**Figure 2:**
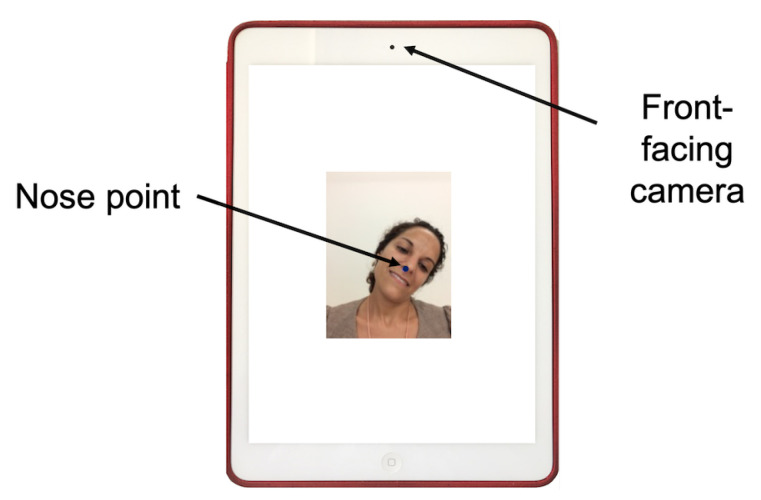
Screenshot of the experiment software with annotations on an Apple iPad Air (device in portrait orientation).

The experiment was conducted on an Apple iPad Air with a front-facing camera placed in the center on the upper side of the device, in portrait orientation. The software executed the head-tracker interface and showed on the screen the image captured by the front-facing camera with the nose point returned by the head-tracker marked with a blue circle (see Figure 2).

An important added value of using the exergame is to enable the user to perform the exercises alone by means of controlling the correct performance. Therefore, it is very important to consider the system’s feedback to inform the user and to help him or her to control the performance. In this sense, it is necessary to explore the neck range of motion (ROM) that could be effectively tracked by the camera-based head-tracker, as a feasibility indicator for neck rehabilitation purposes.

Each participant performed the session in an experiment room, seated in upright position, resting their arms on a table. The iPad with the camera-based head-tracker running, was placed in front of their face at approximately 37 cm, a distance equivalent to naturally holding the device by the subject, with the top line of the iPad screen at eye level [34].

**Figure 3:**
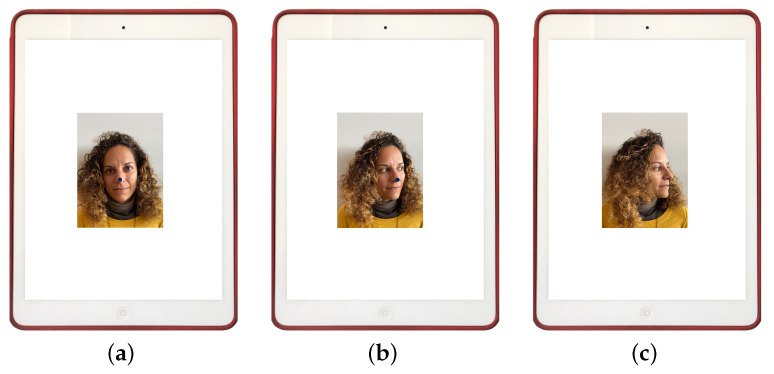
Simulated stages of the experiment procedure for the left rotation movement on the apparatus: (**a**) participant placed in the initial position with the nose point returned by the head-tracker marked with a blue circle, (**b**) the participant starts a rotation movement slowly (the nose point returned by the head-tracker is marked with a blue circle), (**c**) moment when the head-tracker loses the position of the nose (an acoustic alert is triggered) and the participant stops its movement.

**Figure 4:**
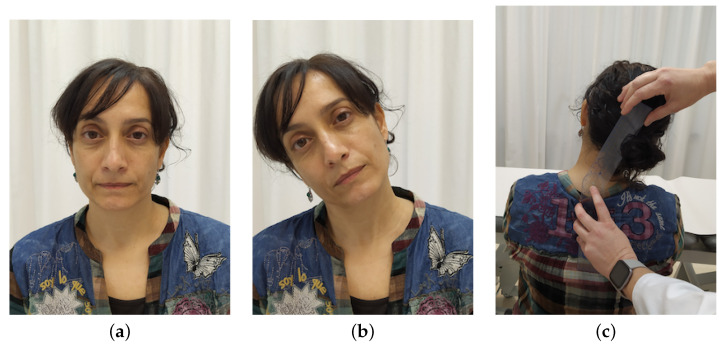
Stages of the experiment procedure: (**a**) participant placed in the initial position, (**b**) participant once the acoustic alert sounded and the participant stopped the movement, (**c**) measurement of the neck ROM of the participant using the goniometer.

**Figure 5:**
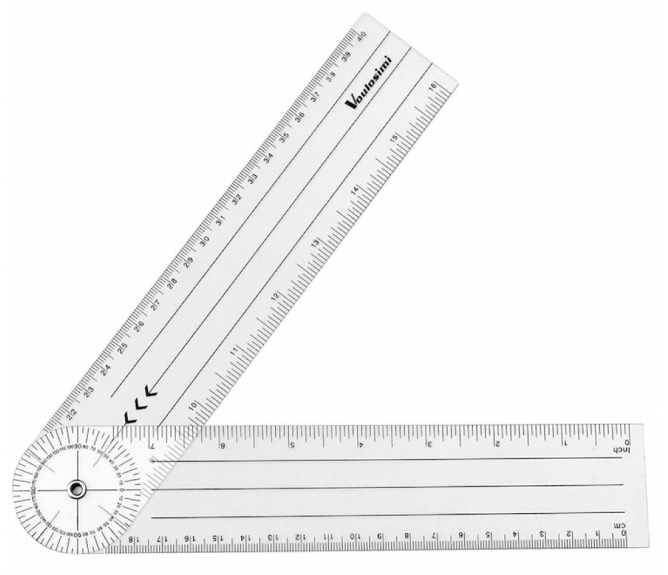
Dual-arm universal goniometer.

They were asked to move their head slowly as far as possible in the direction being studied (flexion, extension, right lateral flexion, left lateral flexion, right rotation and left rotation). See Figure 3. At the moment that the head-tracker lost the position of the nose (see Figure 3c), an acoustic alert sounded and the participant was asked to stop the movement. Then, ROM was measured with a goniometer (see Figure 4c), that is a reliable tool for ROM measurement used in standardized clinical examinations [35,36,37]. It consisted of a stationary arm, a movable arm and a fulcrum or axis, as can be seen in Figure 5.

Flexion and extension were measured with the axis over the external auditory meatus, with the stationary arm parallel to ground and the movement arm in line with the base of nares. Lateral flexion was measured with the axis over spinous process of C7 vertebra, with the stationary arm in line with spinous processes of thoracic vertebrae, so that arm was perpendicular to ground, and the movement arm in line with occipital protuberance. Rotation was measured with the axis located over central aspect of head, with the stationary arm parallel to imaginary line between the two acromial processes, and the movement arm in line with the tip of the nose. All measurements were conducted by an experienced physiotherapist.

Each movement was repeated three times, and equally measured (see Figure 4). Testing lasted about 15 min per participant.

The total number of measures was 7 participants × 6 directions × 3 repetitions = 126.

In Table 1, mean and standard deviation values, as well as proportion of normal maximum ROM tracked, are given for each movement direction of the cervical area (flexion, extension, right lateral flexion, left lateral flexion, right rotation, left rotation).

**Figure 6:**
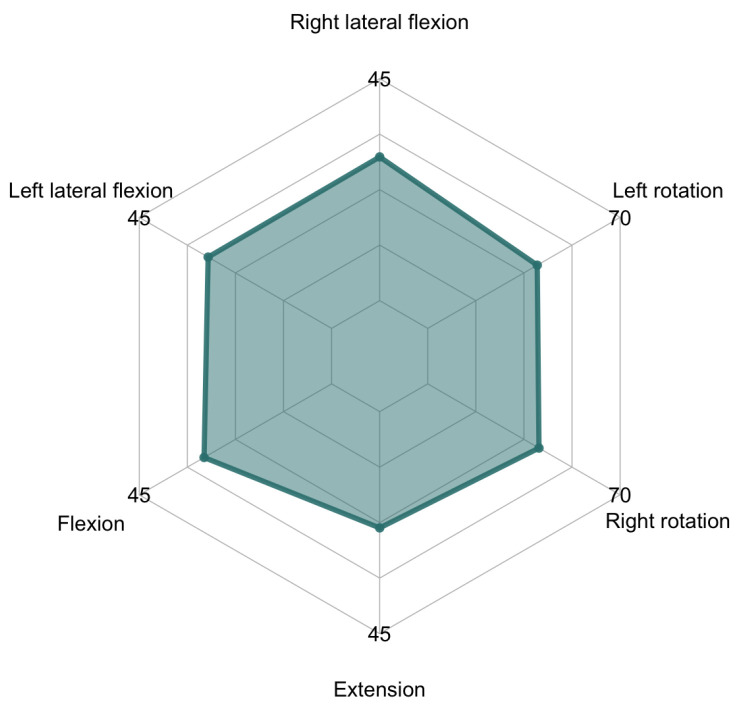
Mean values of the neck range of motion (ROM) tracked by the head-tracker interface compared to normal maximum mobility.

As it is shown in Table 1 and Figure 6, the camera-based head-tracker was able to track the nose position from 50% to 64.5% of the normal maximum cervical mobility ranges. The better detected movements were flexion and lateral flexion, that is, the movements where nose is more visible for the front-facing camera during the user’s movement. Extension implies that nose would be seen in a different position as movement advances, so the head-tracker would lose the nose position at about 23${}^{{}^{\circ}}$ of extension. As for rotation, the head-tracker was able to track the nose position up to about 40${}^{{}^{\circ}}$, moment where the nose was no longer visible from the front by the front-facing camera.

These results proved that the camera-based head-tracker could track the nose position within functional ROM limits, so it could be useful for the rehabilitation of functional mobility, as well as other parameters of rehabilitation (speed of movement, motor control, oculocervical dissociation) within these ranges. This conclusion led us to the design of a mobile application with an exergame that would aim to rehabilitation of functional movement and motor control.

## 3. Validation of the Mobile Application for Monitoring the Neck Movements

Once it was proved that the camera-based head-tracker was feasible for rehabilitation purposes, in the matter of monitoring functional mobility, the application started to be designed. One of the first steps was to determine its safety, in terms that the software must not require more ROM, rapid or complex movements than patients were able to perform at that moment, as they could cause harm and be counterproductive for patients’ recovery. Therefore, a safety validation was needed.

### 3.1. Materials and Methods

For this first safety experiment we focused on the ROM that the software required, because demanding more movement that a patient was able to perform, or a movement that reproduced pain, was considered a potential source of unsafe situations. We would consider the application as safe, in this sense, if the movements asked for the application and therefore performed by the subject in each direction were within the maximum ROM that a patient was able to achieve with no pain. As it was expected to be safe for different patients with different capabilities, or the same patient at different moments of his or her recovery, it was considered necessary to study safety with different capability profiles.

In order to do that, different patient preset profiles were designed. They were designed by experienced physiotherapists, based on general clinical evolution of ROM and neck pain data in actual patients [38,39]:

- Profile 1: corresponded to a low cervical mobility person with or without pain associated with movement.
- Profile 2: corresponded to a lower-medium cervical mobility person, with less or no pain associated with movement.
- Profile 3: corresponded to an upper-medium mobility person and no pain.
- Profile 4: was supposed to represent a patient with an advanced recovery situation or a healthy subject, with normal mobility and no pain.

Range of motion preset for each profile can be seen in Table 2.

**Figure 7:**
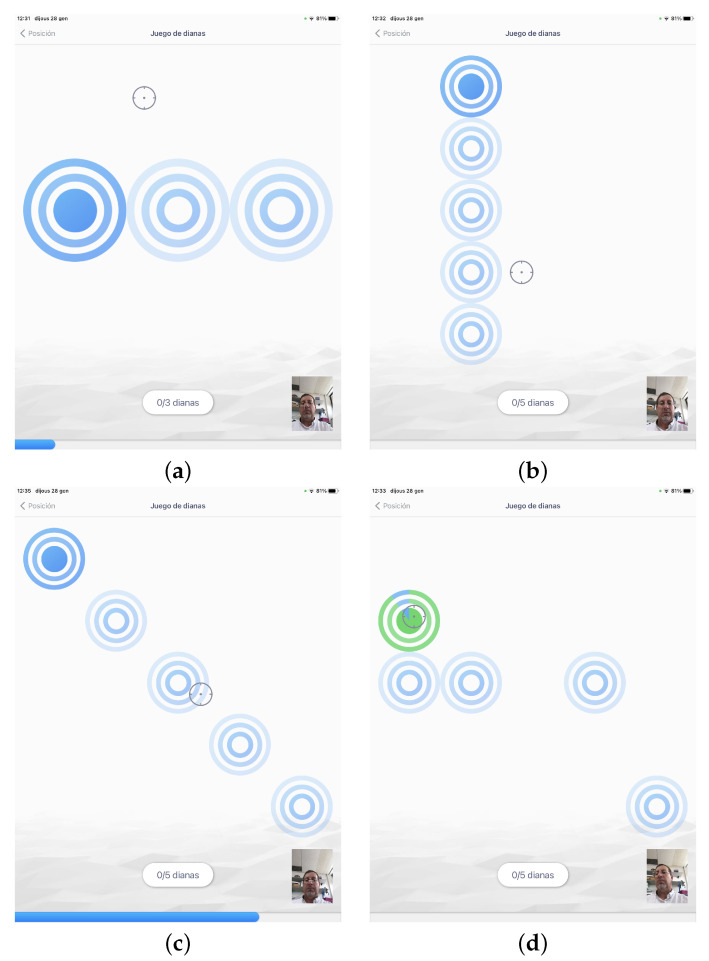
Examples of exercises for every preset profile: (**a**) dartboards set to induce a simple horizontal movement (rotation) with big targets, (**b**) dartboards set to induce a simple vertical descending movement (flexion) with small targets, (**c**) dartboards set in diagonal to induce a combined movement with small targets, and (**d**) dartboards set randomly.

We designed an exergame simulating a dart game with targets to be selected using the movement of the head (see Section 3.1.2 for further description of the game). The configuration of target size, target location, order of targets, time of appearance and disappearance of the target, gain of the head-tracker and dwell-time criterion for selection were set differently for each preset profile:

- Profile 1: required horizontal, vertical or diagonal simple movements with low ROM, with no speed or time pressure. So, configuration for this profile consisted in offering big, easy targets available all the time, that should be selected in a predetermined order, with a high gain factor and no dwell-time. As an example, to require a simple right rotation movement, three big targets available at the same time, that should be selected left to right, were shown on the screen (see Figure 7a).
- Profile 2: the group of exercises included for this profile were those for profile 1 plus exercises with smaller targets, complex movements, and higher speed. These exercises configuration implied more and smaller targets, and a sequential appearance and disappearance of targets, in the preset order of selection, as to introduce a speed factor. As examples of these exercises, Figure 7b shows the location of targets in a simple flexion movement, starting with the upper target, and Figure 7c shows a combined movement: a diagonal.
- Profile 3: the group of exercises loaded for this profile were those with smaller targets, complex movements, movements at a higher speed and motor control requirements. To do so, targets would appear in a random location, with preset time of appearance and disappearance, and with dwell-time to require steadiness in target selection (see an example exercise in Figure 7d).
- Profile 4: the group of exercises loaded for this profile were provided with small targets, complex randomly required movements at a high speed, and motor control requirements. It did not imply more ROM than the previous profiles, as it focused in other parameters (speed, reaction time, motor control). The configuration was similar to profile 3 exercises, but with smaller targets, less time available on screen, less gain factor and more dwell-time on those with motor control requirement.

For the application to be considered as safe, it was supposed to solicit movements within the maximum ROM preset for each profile (see Table 2). In order to validate that the application induced the user to correctly perform the neck movements for every preset profile, it was required that the performed user neck movements were within 80th percentile of the ROM preset for each movement in the different profiles.

#### 3.1.1. Participants

A total of 24 asymptomatic participants (12 females) were recruited from staff and postgraduate students from a university campus in Spain. To be included, participants had to be aged 20 to 64. The average age was 34 years (SD = 13.06). They were excluded if they had reported or complained of neck, shoulder, and/or head impairments or had pain in the preceding month.

#### 3.1.2. Apparatus

The experiment was conducted on an Apple iPad Mini 4 due to ergonomic reasons related to the device’s weight. All communication with the mobile device was disabled during testing.

**Figure 8:**
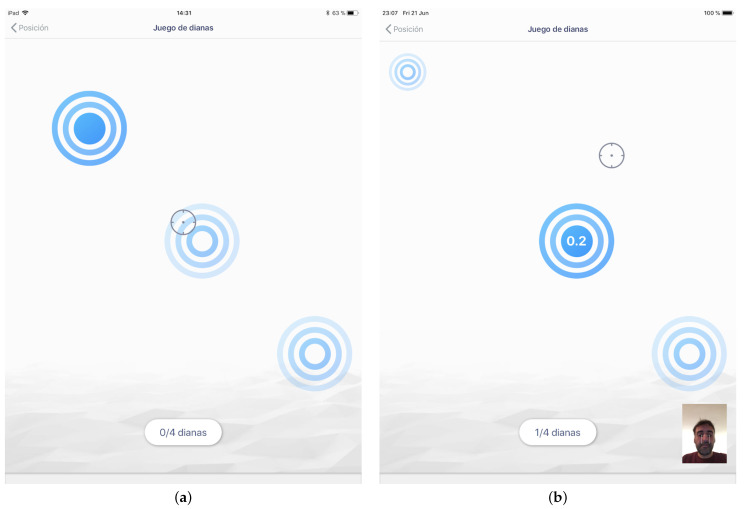
Screenshots of the dart game with two dwell-time criterion: (**a**) 0-ms dwell-time criterion: the target was selected immediately when the center of the cursor entered inside the target, and (**b**) 200-ms dwell-time criterion.

The software simulated a dart game (see Figure 8 for details). The study involved a simple pointing task where the targets to be selected simulated dartboards of different sizes. User input combined the mobile head-tracker for the pointing action and a dwell-time criterion for selection.

The application started with a login form, which was used to determine the patient profile. According to the profile that was established, the application showed different target patterns covering the desired range of motion of the profile (following either horizontal, vertical, diagonal or random paths). The user got a visual feedback of its motion through the movement of a cursor displayed in the dart game.

**Figure 9:**
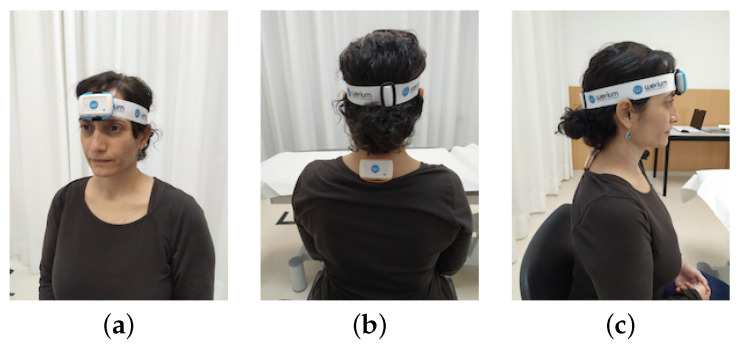
Placement of the inertial sensors: (**a**) sensor 1 (**b**) sensor 2 (**c**) sensor 1 and sensor 2.

To measure the real-time neck mobility performed by subjects while using the application, we used wireless ENLAZA inertial sensors (Werium™system [40]). Inertial sensors included an accelerometer, a gyroscope and a magnetometer which sensed linear acceleration, the turn rate and an accurate angular monitoring along three reference axis. Inertial sensors are widely used to measure motor activities [41,42,43]. Specifically, Werium intertial sensors were previously validated for cervical ROM as well as for other joints [44,45]. The sensors are small and lightweight, so they are barely invasive. Their use would allow to record full ROM, as we previously stated that the application itself was not able monitor full ROM. Two sensors were placed on the subject to conduct the real-time measurement, following the location protocol given by the manufacturer. Sensor 1 was placed on the forehead of the subject, fixed with an elastic band (see Figure 9a,c). Sensor 2 was placed on T1-T2 thoracic vertebrae, fixed with double sided fixing tape (see Figure 9b,c).

#### 3.1.3. Procedure

The study was conducted according to the guidelines of the Declaration of Helsinki and approved by the Research Ethics Committee of the University of the Balearic Islands (Exp.174CER20). Each experiment was conducted by a collaborator of the research team, with previous training on the procedure. The experiment consisted in performing several games/matches with the exergame, corresponding to different levels of demand of ROM.

**Figure 10:**
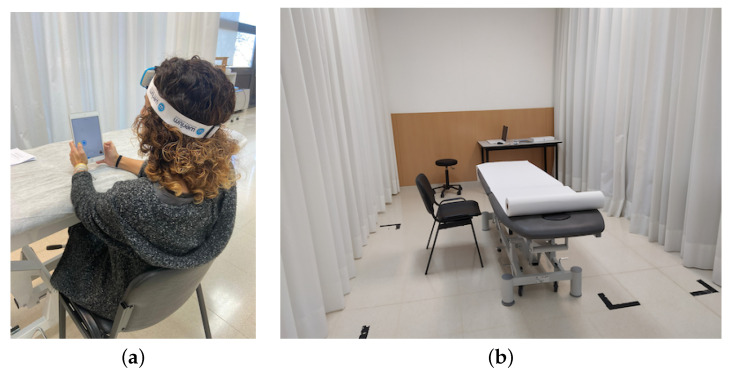
(**a**) Subject of the experiment playing the exergame in (**b**) the experiment room.

Informed consent was obtained from all subjects involved in the study. They were instructed to play the exergame, following the application instructions, holding the device still and moving the cursor by moving their head. Subjects were in an experiment room, seated in a chair with their back supported on the backrest, naturally holding the iPad in front of their head (see Figure 10), with their elbows resting on a height-adjustable treatment table. This position (back on the backrest and elbows resting on the table) was designed to maintain a natural position holding a mobile device while controlling possible compensation movements.

After sensors were placed, subjects were asked to play the exergame, and to select targets as quickly and accurately as possible. Every subject was asked to play a match with the exergame set for each profile. They were allowed to rest as needed between profiles. Movement in every direction was real-time recorded via the inertial sensors and Werium software. Any incident observed or informed by the subjects was recorded by the collaborator. Testing lasted about 30 min per participant.

The total number of sessions was 24 participants × 4 profiles = 96. During every session, the ROM of the subjects reported by the sensors (flexion, extension, lateral flexion, rotation) were recorded.

## 4. Results and Discussion

In this section, results for each preset profile are given for all the movement directions of the cervical area: flexion, extension, right lateral flexion, left lateral flexion, right rotation, left rotation.

Outliers were removed using the Interquartile Range (IQR) method.

Results showed that recorded mobility was below the 80th percentile of the established maximum at all movements and profiles. Even the maximum movement measured stayed within limits in almost every movement and profile, except for extension. Extension movements in profiles 1 and 2 (see Table 3 and Table 4) showed that the maximum recorded exceeded significantly the preset limits at least once. One possible explanation, from the observation and data, suggested that the maximum of extension recorded may be due to moments in which the head-tracker reference was lost, and the manoeuvre done by the subject to recover the nose tracking was doing a movement out of range of the front-face camera and back, that could be an extension movement. Nevertheless, extension movement was within limits at 80th percentile for these profiles. For profiles 3 and 4 (see Table 5 and Table 6), the mobile application would not be able to exceed the limits of ROM preset due to the head-tracker limits that were determined in the first experiment.

Results not only confirmed safety, in terms of ROM requirements for each profile, according with the safety parameters previously established, but informed also about other aspects. One of these findings was that the mobile application required similar maximum ROM for the different profiles, with differences in flexion: profiles 1, 2 and 3 showed a slightly increase in mobility demand by the mobile application in flexion. Profile 4 (see Table 6), on the other hand, did not show this increasing tendency. Profile 4 was not preset as to continue this increase tendency, but to focus on other rehabilitation parameters. Thus, it was also expected not to detect an increase of ROM demand for this profile. In addition, one unexpected finding was the virtual absence of lateral flexion for the interaction with the mobile application, observed in every profile. Further work is required to clarify whether it is due to the configuration of the targets on the screen (and so, a mobile application performance finding), or whether it is a phenomenon linked to the interaction with mobile devices in general.

All these findings, in addition to the results of the intended evaluation, provided valuable information for future lines of work. Data collected suggested that it would be the combination of different parameters of the mobile application, and not only ROM, what would determine the effectiveness of the exergame as a therapeutic exercise application. In order to work gradually and adapt to the objectives of the rehabilitation and the patient’s situation (functional mobility recovery, motor control, strength, etc.), it would be needed to combine parameters as required speed of movement, dwell-time for target selection, number of targets, moment of appearance or disappearance of the targets, etc. If we want to focus on more ROM demand for the medium and advanced profiles (namely profile 3 and 4), it would be necessary to study the feasibility of working with the gain parameter, so the mobile application would demand more ROM for the same movement in screen.

## 5. Conclusions

In this paper, we present a evaluation study of a camera-based head-tracker mobile application for monitoring neck movements for cervical rehabilitation purposes. Due to the use of the camera, this application has the advantage that it does not need any contact sensor and the user can move freely, without any external limitation. The objective of this paper is to present the technical feasibility and a first safety evaluation of the camera-based head-tracker to monitor the neck movements.

First, we explored at what extent the head-tracker was able to monitor the user’s neck movements. On that question, the study found that camera-based head-tracker is able to monitor from the 50% to the 64.5% of the full cervical range of motion. This result proved that a camera-based head-tracker was a feasible tool for cervical rehabilitation purposes, within a functional mobility range of motion. Secondly, an exploratory validation experiment has determined the safety of the designed head-tracker mobile application. We checked that is possible to use the head-tracker for neck movement detection in an exergame for cervical rehabilitation purposes and, that is safe in terms of the required neck range of motion demanded by the exergame for each preset patient profile. At this point, we can conclude that the camera-based exergame did not require more range of motion than the preset profile user was capable to perform.

As the objective of this paper was to present the technical feasibility and a first safety study, participants in the presented studies were healthy subjects. In order to continue with the validation of the exergame mobile application using a camera-based head tracker as a sensor, further work needs be conducted, including clinical research studies, with actual patients.

In addition, we point out that this research could serve as a base for future studies on the use of camera-based head-trackers for other healthcare purposes, as we proved that is able to monitor the user’s head movements by means of the front-facing camera of a mobile device.

## Figures and Tables

**Table 1:** Mean values and standard deviation of cervical range of motion measurements, and their relationship with normal mobility range.

Movement	ROM Detected	Normal Maximum ROM	% of Maximum ROM Tracked
Flexion	29.05${}^{{}^{\circ}}$ ± 6.25${}^{{}^{\circ}}$	45${}^{{}^{\circ}}$	64.55%
Extension	22.86${}^{{}^{\circ}}$ ± 6.63${}^{{}^{\circ}}$	45${}^{{}^{\circ}}$	50.79%
Right lateral flexion	27.39${}^{{}^{\circ}}$ ± 4.64${}^{{}^{\circ}}$	45${}^{{}^{\circ}}$	60.85%
Left lateral flexion	28.10${}^{{}^{\circ}}$ ± 3.70${}^{{}^{\circ}}$	45${}^{{}^{\circ}}$	62.43%
Right rotation	39.05${}^{{}^{\circ}}$ ± 6.25${}^{{}^{\circ}}$	70${}^{{}^{\circ}}$	55.78%
Left rotation	38.81${}^{{}^{\circ}}$ ± 6.70${}^{{}^{\circ}}$	70${}^{{}^{\circ}}$	55.44%

**Table 2:** ROM of the preset profiles.

Profile	Flexion	Extension	Lateral Flexion	Rotation
Profile 1	20${}^{{}^{\circ}}$	15${}^{{}^{\circ}}$	5${}^{{}^{\circ}}$	25${}^{{}^{\circ}}$
Profile 2	30${}^{{}^{\circ}}$	20${}^{{}^{\circ}}$	35${}^{{}^{\circ}}$	40${}^{{}^{\circ}}$
Profile 3	40${}^{{}^{\circ}}$	40${}^{{}^{\circ}}$	45${}^{{}^{\circ}}$	70${}^{{}^{\circ}}$
Profile 4	45${}^{{}^{\circ}}$	45${}^{{}^{\circ}}$	45${}^{{}^{\circ}}$	70${}^{{}^{\circ}}$

**Table 3:** ROM results of Profile 1 (After outlier filtering, the analyzed data for the Profile 1 supposed a 98% of the total flexion-extension raw data, a 96% of the total lateral flexion raw data, and a 99% of the total rotation raw data). The cases where the maximum ROM of the subjects reported by the ENLAZA inertial sensors exceeded the preset ROM defined for the profile are marked with an asterisk.

Movement	Mean	Standard Deviation	Maximum	80th Percentile	Preset ROM
Flexion	4.39${}^{{}^{\circ}}$	3.40${}^{{}^{\circ}}$	19.17${}^{{}^{\circ}}$	6.91${}^{{}^{\circ}}$	20${}^{{}^{\circ}}$
Extension	8.15${}^{{}^{\circ}}$	5.97${}^{{}^{\circ}}$	26.16${}^{{}^{\circ}}$ *	13.15${}^{{}^{\circ}}$	15${}^{{}^{\circ}}$
Right lateral flexion	2.00${}^{{}^{\circ}}$	1.62${}^{{}^{\circ}}$	6.51${}^{{}^{\circ}}$ *	3.32${}^{{}^{\circ}}$	5${}^{{}^{\circ}}$
Left lateral flexion	1.45${}^{{}^{\circ}}$	1.09${}^{{}^{\circ}}$	5.49${}^{{}^{\circ}}$ *	2.30${}^{{}^{\circ}}$	5${}^{{}^{\circ}}$
Right rotation	7.03${}^{{}^{\circ}}$	5.43${}^{{}^{\circ}}$	23.41${}^{{}^{\circ}}$	11.60${}^{{}^{\circ}}$	25${}^{{}^{\circ}}$
Left rotation	5.71${}^{{}^{\circ}}$	4.21${}^{{}^{\circ}}$	21.51${}^{{}^{\circ}}$	9.34${}^{{}^{\circ}}$	25${}^{{}^{\circ}}$

**Table 4:** ROM results of Profile 2 (After outlier filtering, the analyzed data for the Profile 2 supposed a 98% of the total flexion-extension raw data, a 97% of the total lateral flexion raw data, and a 98% of the total rotation raw data). The cases where the maximum ROM of the subjects reported by the ENLAZA inertial sensors exceeded the preset ROM defined for the profile are marked with an asterisk.

Movement	Mean	Standard Deviation	Maximum	80th Percentile	Preset ROM
Flexion	9.18${}^{{}^{\circ}}$	6.84${}^{{}^{\circ}}$	30.98${}^{{}^{\circ}}$ *	14.46${}^{{}^{\circ}}$	30${}^{{}^{\circ}}$
Extension	7.74${}^{{}^{\circ}}$	5.77${}^{{}^{\circ}}$	26.75${}^{{}^{\circ}}$ *	12.70${}^{{}^{\circ}}$	20${}^{{}^{\circ}}$
Right lateral flexion	2.13${}^{{}^{\circ}}$	1.55${}^{{}^{\circ}}$	7.10${}^{{}^{\circ}}$	3.33${}^{{}^{\circ}}$	35${}^{{}^{\circ}}$
Left lateral flexion	1.78${}^{{}^{\circ}}$	1.39${}^{{}^{\circ}}$	6.29${}^{{}^{\circ}}$	2.85${}^{{}^{\circ}}$	35${}^{{}^{\circ}}$
Right rotation	6.50${}^{{}^{\circ}}$	4.92${}^{{}^{\circ}}$	21.51${}^{{}^{\circ}}$	10.83${}^{{}^{\circ}}$	40${}^{{}^{\circ}}$
Left rotation	5.48${}^{{}^{\circ}}$	4.24${}^{{}^{\circ}}$	20.00${}^{{}^{\circ}}$	8.79${}^{{}^{\circ}}$	40${}^{{}^{\circ}}$

**Table 5:** ROM results of Profile 3 (After outliers’ filtering, the analyzed data for the Profile 3 supposed a 98% of the total flexion-extension raw data, a 93% of the total lateral flexion raw data, and a 99% of the total rotation raw data).

Movement	Mean	Standard Deviation	Maximum	80th Percentile	Preset ROM
Flexion	10.16${}^{{}^{\circ}}$	7.87${}^{{}^{\circ}}$	33.37${}^{{}^{\circ}}$	16.96${}^{{}^{\circ}}$	40${}^{{}^{\circ}}$
Extension	9.01${}^{{}^{\circ}}$	7.02${}^{{}^{\circ}}$	30.22${}^{{}^{\circ}}$	14.78${}^{{}^{\circ}}$	40${}^{{}^{\circ}}$
Right lateral flexion	2.11${}^{{}^{\circ}}$	1.81${}^{{}^{\circ}}$	7.30${}^{{}^{\circ}}$	3.65${}^{{}^{\circ}}$	45${}^{{}^{\circ}}$
Left lateral flexion	2.31${}^{{}^{\circ}}$	1.74${}^{{}^{\circ}}$	8.00${}^{{}^{\circ}}$	3.61${}^{{}^{\circ}}$	45${}^{{}^{\circ}}$
Right rotation	9.00${}^{{}^{\circ}}$	6.48${}^{{}^{\circ}}$	29.51${}^{{}^{\circ}}$	14.67${}^{{}^{\circ}}$	70${}^{{}^{\circ}}$
Left rotation	6.10${}^{{}^{\circ}}$	4.96${}^{{}^{\circ}}$	21.67${}^{{}^{\circ}}$	10.00${}^{{}^{\circ}}$	70${}^{{}^{\circ}}$

**Table 6:** ROM results of Profile 4 (After outliers’ filtering, the analyzed data for the Profile 4 supposed a 99% of the total flexion-extension raw data, a 96% of the total lateral flexion raw data, and a 98% of the total rotation raw data).

Movement	Mean	Standard Deviation	Maximum	80th Percentile	Preset ROM
Flexion	7.05${}^{{}^{\circ}}$	5.16${}^{{}^{\circ}}$	24.84${}^{{}^{\circ}}$	11.47${}^{{}^{\circ}}$	45${}^{{}^{\circ}}$
Extension	8.70${}^{{}^{\circ}}$	6.16${}^{{}^{\circ}}$	29.67${}^{{}^{\circ}}$	14.12${}^{{}^{\circ}}$	45${}^{{}^{\circ}}$
Right lateral flexion	1.68${}^{{}^{\circ}}$	1.27${}^{{}^{\circ}}$	6.15${}^{{}^{\circ}}$	2.71${}^{{}^{\circ}}$	45${}^{{}^{\circ}}$
Left lateral flexion	1.96${}^{{}^{\circ}}$	1.58${}^{{}^{\circ}}$	6.20${}^{{}^{\circ}}$	3.39${}^{{}^{\circ}}$	45${}^{{}^{\circ}}$
Right rotation	6.40${}^{{}^{\circ}}$	4.80${}^{{}^{\circ}}$	22.07${}^{{}^{\circ}}$	10.37${}^{{}^{\circ}}$	70${}^{{}^{\circ}}$
Left rotation	6.25${}^{{}^{\circ}}$	4.67${}^{{}^{\circ}}$	20.70${}^{{}^{\circ}}$	10.32${}^{{}^{\circ}}$	70${}^{{}^{\circ}}$

## Data Availability

The data presented in this study are available on request from the corresponding author. The data are not publicly available due to data privacy restrictions.
